# Leveraging qualitative approaches to guide sustainable international research collaborations

**DOI:** 10.1371/journal.pgph.0002941

**Published:** 2024-03-19

**Authors:** Roxanne Vandermause, Rachel Kryah, Julie Bertram, Hannah L. Stewart, Nil Ean, Steven Bruce, Adam W. Carrico, Julie A. Mannarino, Robert H. Paul

**Affiliations:** 1 College of Nursing, University of Missouri–St. Louis, St. Louis, Missouri, United States of America; 2 Missouri Institute of Mental Health, University of Missouri–St. Louis, St. Louis, Missouri, United States of America; 3 School of Public Health, University of Texas Health Science Center, Houston, Texas, United States of America; 4 The Center for Trauma Care and Research Organization, Phnom Penh, Cambodia; 5 Royal University of Phnom Penh, Phnom Penh, Cambodia; 6 Center for Trauma Recovery, University of Missouri–St. Louis, St. Louis, Missouri, United States of America; 7 Department of Psychological Sciences, University of Missouri–St. Louis, St. Louis, Missouri, United States of America; 8 Stempel College of Public Health and Social Work, Florida International University, Miami, Florida; Rural Women's Social Education Centre, INDIA

## Abstract

Qualitative research approaches were used to launch an international research collaboration between the U. S. and Cambodia. Cambodian officials requested assistance in learning qualitative approaches to complement the research skills of Cambodian mental health providers. This article provides a description of how U. S. researchers responded to that request and engaged with Cambodian psychiatrists to explore mental health needs and interventions in both countries and initiate a sustainable relationship. The early focus on qualitative research methodologies may be an avenue that mitigates some of the challenges that can characterize international research. In this study, early communications involved developing a plan to teach qualitative methods while also collecting and analyzing data in both countries that would address the mental health concerns experienced by respective care providers. A case study exemplar was embedded with a scripted focus group guide to collect data from U. S. focus groups, then share with Cambodian psychiatrists. Components of hermeneutic phenomenological interviewing and descriptive content analysis were used to simultaneously teach and enact the research methods, gather data in both countries to analyze, and inspire participants to replicate the methods in their ongoing work. Cambodian psychiatrists were able to demonstrate competence in facilitating focus groups after being participant-observers. Researcher/practitioners from both U. S. and Cambodian teams gained new understandings about the mental health needs of their patients. The mutual engagement of a research focus is an effective way to establish cross-cultural relationships. The challenges of staying with stable teams over times remain, but the content shared and learned in a participatory structure yields understandings that cross cultural boundaries. Anticipated and unexpected challenges may be offset by an intention of reciprocity and mutual engagement. The use of qualitative methodologies, early and repeatedly, can facilitate relational understanding.

## Introduction

International research partnerships, especially those involving nations of disparate income levels, provide a foundation for capacity building, the benefits of which extend bilaterally, including gaining knowledge of people and places, testing interventions, delivering evidence-based research interventions, and teaching skills or customs. The complexity of these partnerships and the multi-purpose goals and varied perspectives from multiple stakeholders have potential to catalyze new scientific directions capable of transforming clinical practice, as well as generate unforeseen challenges in the planning, implementation, and dissemination of such projects. In this article, we describe the planning and implementation of an international research and capacity building collaboration between U.S.-based investigators, mental health experts in Cambodia, and the government agencies in Cambodia that oversee the development and deployment of mental health services (Department of Mental Health and Substance Abuse, Department of Mental Health, the Kingdom of Cambodia), referred to herein as project TITAN (Trauma Informed Treatment Algorithms for Novel Outcomes). We focus on one segment of the long-term project related to the integration of qualitative methodologies and methods to inform a subsequent intervention study. The early focus on qualitative research methodologies may be an avenue that mitigates some of the challenges that characterize international research.

Project TITAN is an ongoing research collaborative funded by the National Institute of Mental Health (NIMH; R01MH114722) through the Fogarty International Brain Disorders in the Developing World research initiative. Project TITAN was designed as a partnership between U.S. and Cambodian teams to reduce the mental health treatment gap that is endemic to Cambodia and other resource-limited countries. Mental health disorders have increased worldwide [1], and low-and middle-income countries (LMIC), like Cambodia, are significantly and disproportionately burdened [2]. Cambodia has suffered unique traumas rendered during the Khmer Rouge Regime of the late 1970s [3, 4]. During that time 1.5 million Cambodians died of starvation and illness, 500,000 were executed and two million displaced. This extreme violence and the legacy of trauma resulted in high levels of psychological distress among the general population [5, 6], with more recent/ongoing challenges related to high levels of poverty, interpersonal violence, and substance use in Cambodia [7–9].

Psychiatric services are limited in Cambodia. Educated individuals were among the targeted groups during the Khmer regime. The downstream impact of the political agenda nearly crippled already vulnerable systems of healthcare and academic research. Further complicating the outlook, no medical graduates have selected specialty training in psychiatry since 2012, and the majority of existing psychiatrists are expected to reach retirement age within the next 10 years. The shortage of medically trained providers combined with the magnitude of mental health problems in Cambodia means that a psychiatric-centered response will not meet the needs of the population. Further, relief from nongovernment organizations (NGOs) cannot resolve the challenge. Previous programs initiated by foreign NGOs failed to align funding opportunities and programmed activities with the long-view needs of government and academic leadership in Cambodia, resulting in circumscribed scale-up and inappreciable sustainability. The University Health Sciences in Phnom Penh is the only medical school in the country. Psychiatrists currently educated in Cambodia are English speaking and study western principles of medical care. Thus, there are opportunities for bidirectional learning opportunities, which was the thrust of the current project.

Early conversations with Cambodian officials revealed the continued, high need for mental health services in the country and a desire to partner to address this problem. Researching clinicians in Cambodia were unfamiliar with qualitative methodologies, so U. S. expertise was requested to teach and work alongside Cambodian researchers to develop sustainable skills in this area. The idea was to work collaboratively to teach and use qualitative findings to inform culturally relevant intervention research. Ultimately, a novel mental health intervention for individuals with Post-Traumatic Stress Disorder (PTSD) using insights obtained from the qualitative work was done. The RCT and results are detailed by Mannarino et al., [10] and the research partnership continues.

The partnership was envisioned to be sustainable and of mutual benefit. Investigators from both countries had recently completed a collaborative research effort sponsored by the NIMH (R01MH102151) that focused on neurodevelopment among perinatally acquired HIV youth in Thailand and Cambodia. This successful collaboration resulted in multiple research publications from the international team [11–15] and set the stage to expand the science and capacity building opportunities. The timing was fortuitous as Cambodia was in the process of updating the national guidelines for the provision of mental health service delivery. Outcomes from Project TITAN were envisioned to provide evidence-based insights to assist with the development of the revised national plan for mental health care.

A seminal cohort of five psychiatrists were chosen by the Minister of Health (MOH) to work with the U. S. team to launch the research partnership. The goal was to share best practices in mental health research design, implementation, and analysis and gain critical insights about relevant barriers and facilitators governed by cultural, political, and pragmatic factors unique to Cambodia. Thus, mental health practitioners could be prepared for, not only the dissemination of results of the clinical trial that were part of the project but also, ongoing assessment and development of evidence-based interventions culturally tailored for Cambodian citizens. Furthermore, assessment practices and interventions resulting from the project could improve mental health practices in the U.S.

The proposed work provided the U. S. team an opportunity to integrate the previous work done across core research areas/Departments at the University of Missouri, St. Louis and Miami School of Medicine (precision health, program development and evaluation, professional training, clinical trials) into a singular project with the ultimate goal of shortening the typical time from concept to clinical implementation (~14 years). To hasten the pace of implementation, Project TITAN aimed to move beyond the typical "siloed" approach to clinical research by simultaneously leveraging critical insights from multidisciplinary experts involved in discrete (and typically, encapsulated) stages of work relevant to the continuum of clinical care.

The U.S. team assembled scientists to conduct qualitative and experimental research, both of which were needed to develop and implement a culturally informed treatment for PTSD in Cambodia, and to prepare to engage with the Cambodian team in all aspects of the work. The initial research activity included the development of a process to collect qualitative data while also sharing best practices in qualitative research skills to members from both countries. *It is this emphasis on qualitative methods*, *which engages stakeholders in practical human conversations and focuses on experience over theory*, *that provided a mechanism for interaction that helped us move forward into a long-term reciprocal and sustainable project*.

This article summarizes and interprets the process we used to initiate the research partnership and anchor the qualitative work that guided the overall project.

## International partnerships

International research partnerships are important for improving global health outcomes [16]. These partnerships allow for the pooling of resources, infrastructure, and expertise to implement high-quality, contextually relevant international health research [17]. Additionally, international research partnerships are capable of leveraging a larger, more diverse, and more representative brain trust to address vexing mental health conditions. However, the path to realize these positive outcomes includes challenges that may not be familiar to teams without prior experience conducting international research [16, 18, 19] There has been a longstanding concern that international research partnerships have potential to perpetuate colonial practices- utilizing the host institution’s resources, with unequal recognition related to scientific findings that result from the collaboration [17]. The potential long-term impact on study team personnel residing in the host country is also important to consider. Even when research personnel are compensated highly, these individuals are pulled out of their traditional professional roles, creating a net loss for local systems. Further, it may be difficult for these individuals to re-enter the work force if/when funding ends for a specific project. This creates a double impact (first on the system and then on the individual) and may contribute to the unintended consequences befalling western researchers.

In the most extreme cases, short-term research objectives become prioritized without sufficient interest and/or means to prioritize the long-term needs and interests of all individuals in the partnership [20]. The result is that the research and clinical gap in mental health can persist [21]. At the partnership’s eventual end, the host institution may be no better situated to implement, scale and sustain mental health research and clinical initiatives aimed at reducing the burden of mental health need than when the project started [22]. The vacuum that develops through this extraction of resources has potential lasting consequences that result from a historical understanding that academic and governmental leaders use resources for their own gains and the citizens of the nation themselves are "pawns". That is, institutions in the Global North achieve their own outcomes in the way of external grant funding, scientific publications and professional reputation without considering or contributing to the same in the host country. When such practices are perceived, there is inevitable and understandable resistance in host countries towards establishing de novo research "partnerships".

Such concerns voiced in the literature are difficult to overcome in light of the available funding structures in the U. S. that require reapplications of short-term projects, with extended times between application and acquisition of funds. Further hampering researchers is the manner in which study sections are reviewed by granting institutions. Proposals are evaluated for scientific rigor but may not be as valued on issues of uptake, scalability, and sustainability, matters ultimately determined by government policies that inhibit success. Mental health carries its own complexities and is historically/chronically underfunded by most countries, but this is catastrophically true in most low-and-middle-income countries. Closing the gap requires policy commitments that have not occurred in many of these countries. This makes it difficult to conduct a long-term effective and sustainable outcome. The culture of international research described in the critical literature is important to consider but it need not deter researchers moving toward reciprocity in whatever way is feasible.

A difficulty researchers encounter is that funding systems are not designed to promote long-term partnerships. This is particularly true for Fogarty and is noted in the critical evaluative review of the Fogarty Brain Disorders in the Developing World initiative, but the core issue applies to all of the National Institute of Health institutes and is widely recognized as the primary reason that early career scientists do not succeed. The average time from initial submission to funding is 3 years, which means that "follow-up" grants are submitted in year 2 of the initial 5-year grants. Since the average lag of 3–6 months for project start up is common, and 12 months minimum is typical for publication of findings, there is less than 6 months to collect, analyze, write and disseminate to demonstrate productivity. The Fogarty program is even more problematic. Groups funded with an R21 for two years, must submit the follow-up R01 at the end of the first year of the two-year grant to have any hope of securing the funding that is necessary to maintain the international partnership. The circumstances require innovative and long-term partnership development and the growth of a collaborative team that might change in constellation over time.

In response to these problematic relationships, increased attention has been given to the development of global health partnerships that are characterized by reciprocity [23] with benefits of the partnership shared meaningfully by all stakeholders [24]. The call for reciprocity includes demands for more focus on intentional research capacity building of individuals and organizations via bidirectional learning opportunities [21]. These training activities increase the ability of partner institutions to develop contextually relevant research programs independent of institutions from high-income countries [25]. Our study goals included a robust desire to maintain a long-term collaboration where mutual learning remains a high priority. Considerations of the known challenges of international research guided the goals and processes imbued in this research and the relationship continues today.

## Methodologies and methods

In this research partnership the U.S. team developed a series of didactic workshops to share information about environmental scanning techniques and qualitative research methods to members of the Cambodian team. This was in response to the preliminary needs assessment that indicated a desire for professional development in qualitative research theory, design, implementation, and analysis, all high priorities for the Minister of Health (MOH). Like many U.S. medical schools, Cambodian psychiatrists did not have formal exposure to qualitative methods during their medical training. The U.S. team offered background philosophy and specific techniques for assessing holistic regional and national resources and needs, in lecture and discussion sessions. The emphasis, however, in our on-site educational sessions was on the teaching, learning and implementation of qualitive research methods. An overview of qualitative methods was provided in lecture format along with environmental scanning techniques, but the focus of the initial meetings involved the demonstration and teaching of focus group interviewing, explicated in this article.

The nascent plan for the qualitative U.S. team to establish a connection with the Cambodian team included visiting Cambodia to meet with divisional directors and representatives from the Cambodian MOH, the providers and basic systems of care. We wanted to establish a growing rapport, understand local customs and share personal and professional stories. We ate with one another, travelled to agencies, hospitals and community mental health sites with the MOH on guided tours. We talked around tables to discuss the didactic teachings and plan our research. We wanted to conduct qualitative focus group interviews in Cambodia to assess Cambodian psychiatrists’ experiences treating mental health problems. The U.S. team also prepared to teach qualitative research methods so that the Cambodian psychiatrists could assess and evaluate treatment interventions over time and in the future. Since the proposed RCT for PTSD would challenge existing treatment paradigms, with the long-term plan to implement the method in the U.S., it was important to obtain input from a diverse group of stakeholders in a region of the USA where the mental health treatment gap is large and increasing. Therefore, we needed to identify healthcare characteristics and qualitative research processes in the U.S we could present efficiently during our visit; that would provide an opportunity to compare perceptions of U.S. and Cambodian stakeholders related to the proposed treatment.

This comparison was critical to help inform how learnings obtained in Cambodia could help resolve mental healthcare challenges in rural areas of the U.S. and among individuals who are marginalized from healthcare access even within urban areas. Thus, we conducted a series of structured focus groups in the U.S. so that the U.S. team could develop and practice methods that could be taught and sustained overseas, as well as learn similarities and differences about and with our Cambodian colleagues.

### Development of the focus group plan

Three qualitative researchers, with backgrounds in evaluation research and hermeneutic phenomenology developed a protocol for recruiting, interviewing, analyzing and teaching focus group methods. The methodology included a variety of qualitative approaches useful for particular operational methods. Components of hermeneutic phenomenology, case study, and focus group methods were incorporated into the research plan and subsumed in an overall hermeneutic foundation. Hermeneutic phenomenology is a way of thinking and a technique for interpreting meaning through a dialogic process of participant storytelling. It is, in essence, an attempt to understand and make sense of experience(s) [26]. The rationale for using hermeneutic phenomenology as an overall framework was that it provided an opportunity for participants to express often overlooked or undisclosed matters. This was particularly useful in the Cambodian culture where complex hierarchical systems related to occupational rank, socio-economic status, and gender could inhibit expression. Analysis of text and interpretive team dialogue then led to new understandings not otherwise anticipated. A phenomenological influence in our methods allowed us to engage our Cambodian colleagues in conversations that induced meaningful discussions.

Data collection through focus group interviews was elicited with a reflective opening so participants could take the lead, giving them an opportunity to select what was meaningful or significant. We took cues from the participants about how to proceed. The manner in which the interviews were conducted gave way to analysis or understanding as their stories unfolded [27]. Therapeutic benefits versus harms of various treatment modalities can be elicited during phenomenological interviews; safety and wellbeing are prioritized over the research endeavor to mitigate risks. Meaning is negotiated in a co-constructed narrative account [26].

A hermeneutic approach often uses very broad and unstructured questions, but the methodology is complex. In this study, the need for a more deductive approach that could be understood across groups and replicated, resulted in choosing qualitative descriptive influences [28] and focus group methods [29] using case study interrogatives. Interview questions were designed using case studies. This approach was intended to integrate the hermeneutic and qualitative descriptive influences in that the use of narrative scenarios allowed participants to engage experientially, though the questions were structured.

Focus groups are a participant-driven qualitative research approach in which a group of people, facilitator (s), and a research associate or assistant join to discuss a clinical or research question, concern or concept [29]. The size of the focus group varies but is typically comprised of 7–10 participants (sans study team personnel). Sampling can be homogeneous or heterogeneous and is purposeful. In the case of international research, the composition of the focus group requires understanding of customs relative to rank, age, sex and other characteristics. The cultural habits of disclosure may vary, the spoken language and its nuances may be misunderstood. We needed to account for these challenges in our processes and recognize our unavoidable naivete. This required frequent conversations among all stakeholders.

The team deliberated on who to recruit for the focus groups to test the process and establish the practical implementation that would be practiced, documented and used in the teaching process. The focus groups that were conducted in the U.S. allowed the researchers to begin thinking about clinical perspectives and prepare for comparison elicitations in Cambodia. Procedural challenges could also be identified. Groups were comprised of nurses who worked with individuals with mental health needs (*n* = 9), individuals who are currently receiving mental health care (*n* = 10), and individuals receiving general health care (*n* = 6), for a total of 25 participants (Table 1).

**Table 1:** Focus group participation by groups.

**Group**	**Dates Conducted**	**Location**	**# Participants**
**Group of nurses who work with persons with mental health needs**	November 14, 2017	University College of Nursing	9
**Group of recipients of mental health care**	November 27, 2017	Certified Community Behavioral Health Organization	10
**Group of recipients of general health**	November 18, 2017	University College of Nursing	6
**TOTAL**			**25**

Case study is one method used in focus groups. A case is a bounded system that can be studied to understand its particularities and situatedness [30, 31]. Typically, there is something unique or of value within the case. In our work we began our questioning by using a case study or scenarios crafted by Dr. Brooks, who had worked with the Cambodian therapists for some time prior to our arrival in Cambodia. Common presentations, such as situations involving substance use, manifestations of depression and anxiety and family struggles were created to present to focus group participants for discussion. This strategy ensured we were engaging the participants in practical situations to elicit experiential rather than theoretical expressions of their stories [30]. Analysis of dialogues, description of co-existence of experiences, and study of contexts help refine theory, suggest complexities for further investigation and help establish limits of generalizability. The use of context adds value to the case study approach. Triangulation and thorough description throughout the study add to the rigor. Therefore, the case study process was chosen for the interviews.

#### Data collection

Data collection via qualitative interviews took place at the onset of this long-term partnership as part of the teaching/learning activities. Methods are detailed below. Recruitment for U.S. focus groups began November 1, 2017 and ended with the final focus group conducted by Cambodian researchers on August 13, 2018.

#### Focus groups in the U.S.: Preparation for professional training activities

The focus groups were conducted in November 2017. A group of nurses who worked with patients with mental health needs, a group of recipients of mental health care and a group of recipients of general health care were invited to participate. Participants were recruited through the University College of Nursing and a local Certified Community Behavioral Health Organization. Table 1 shows the group categories and details the time, place and number of participants, to illustrate the composition of the U.S. groups. Participants were compensated for their contributions with a $20 gift card and food was provided. As previously noted, the goal was to conduct focus group interview sessions using the planned methods to develop procedural expertise that would then be shared and taught in Cambodia. The focus group protocol and questions were discussed with the Cambodian team and then approved by the Institutional Review Board at the University of Missouri-St. Louis.

Considerations during the focus group sessions included the arrangement of a setting that was comfortable for participants, allowing them to sit with some distance from one another but close enough for conversation and the assurance that they would be heard. We established ground rules of respect, mutual listening and confidentiality within the group. We expected that by bringing participants together to talk with each other, ideas, attitudes and feelings would be freely generated among the group. Documents and written scripts were prepared ahead of the focus group interview sessions. The purpose of the focus groups was to learn about how members perceived mental health needs in the community. We wanted to know opinions and beliefs about culture, religion, community and family responses to mental health, specifically about depression and anxiety. Participant demographic characteristics, such as profession, age, sex, and educational level, were elicited. One researcher read the script, which described the study purpose and ground rules for the group, and the other two researchers served as notetaker and observer. Verbal consent was obtained to participate and to record the session for transcription purposes. Consent forms were signed and collected. Participants were asked to think about the case study scenarios and respond conversationally, listening to one another and allowing for various participants to have opportunities to talk.

Case studies consisted of a variety of scenarios that told a story about an individual and his symptoms. An example of a case study is represented in Table 2 (Bob’s story). Bob’s story was read to the group and also delivered in hard copy for reference. Focus group members began their discussions based upon Bob’s story and this opening question, “What do you think is going on with Bob?” This approach was intended to elicit experiential responses in the participants, allowing them to associate the abstract inquiry with their own recollections and understandings of similar situations. This hermeneutic approach was intended to keep participants in their storied experiences rather than focusing on cognitive ideas that could distance them from the phenomena discussed.

**Table 2:** Bob’s story.

**Opening Vignette:** Bob is a 50-year old male who has been feeling unwell in the past month. He has daily crying spells and is feeling sad and down “all the time”. He has trouble sleeping at night and it takes him several hours to fall asleep; some nights he doesn’t sleep at all. He spends the majority of his time alone worrying and thinking about his life. He no longer meets with his girlfriend and doesn’t respond to his brother’s calls; he used to enjoy having them all around. He is undereating since nothing tastes good anymore and his weight went significantly down in the past month. He lost interest in his work and already missed seven workdays, but he “doesn’t care anymore”. All he wants is to “do something to run away from this” but he feels there is nothing he can do and no one can really help him.	
**Questions**	**Additional Topics for Questioning** (if not revealed in previous inquiries)
1. What do you think is happening to Bob? What is going on with him?	1. Please describe the most common mental health needs in your community?
2. What are some problems he may have?	2. What treatments are available for mental health needs in your community?
3. What would he do for these problems?	3. How effective are mental health treatments?
4. What kind of help would he need?	4. How does religion and culture influence individuals’ beliefs about mental health?
5. Where would he go to get help?	5. What are the most common symptoms of the primary mental health concerns in your community?
6. Who would help him?	6. How do individuals affected by a mental health condition describe the symptoms?
7. What kind of help would he get?	7. What are some potential barriers to use of non-medication treatment of mental health needs in your community?
8. How does the help you are describing work? Is that help successful? What are some things that may get in the way of it working?	8. What could support use of non-medication treatment of mental health needs in your community?
9. What else can Bob do?	9. Who is qualified to provide non-medication treatment of mental health needs?
10. Is there other help Bob may consider? A different type of person to help?	10. Are community members able to provide effective non-medication treatment of mental health needs?
11. What other mental health problems do people have? What goes along with these problems?	11. Do you believe those diagnosed with a mental condition would feel comfortable receiving non-medication treatment of mental health needs from someone who is not a medical doctor?

The facilitators took on specific roles and responsibilities during the session. The responsibilities consisted of developing the research interview guide, gathering consent, establishing and delivering ground rules at the start of each session, and ensuring the research process is conducted ethically. The facilitators worked in tandem with one taking primary responsibility for encouraging discussion and one observing, writing observations and recording the interaction. The sessions were audio-recorded, transcribed, then cross-checked for accuracy.

A hermeneutic influence was enacted stylistically in the questioning process and later in the analytical components. One researcher facilitated the discussion by walking around the room, gesturing for response when participants indicated they had something to say, asking for elaboration when appropriate and repeating participant responses for clarification when needed. The other researchers took notes and monitored participants who were observing, ensuring all participants were heeded and assessing for discomfort or disagreement through body language. Each focus group session took approximately one hour to complete. A debriefing with the notetaker, observer and researcher was held after each focus group.

#### Focus groups in Cambodia

The Cambodian team consisted of practicing psychiatrists educated in Western-style psychiatric medicine at the University Health Sciences in Phnom Penh. However, the team members were interested in learning qualitative research methods that had not been part of their medical education. Therefore, the U.S. researchers delivered didactic sessions that included the background and processes used in the U.S. focus groups, which were described in detail. Sample documents (interview guides, ethical board review forms, analysis steps, research resources) were shared. Then, the U.S. researchers demonstrated the focus group interview process. The Cambodian team provided insight and feedback about cultural relevance and pragmatic issues related to implementation of the process. They discussed their own plan and then demonstrated a focus group session conducted by several members of the Cambodian team in English, using other members of the Cambodian research team (n = 8) as participants. This session was observed and evaluated by the U.S. team for methodological technique, in oral and written form and discussed among all researchers on the project.

Following the completion of this initial in-person learning collaborative, the U.S. team continued to meet with the Cambodian researchers via online video (Zoom) on a monthly basis to discuss methods and answer questions as the Cambodian team developed their research plan. The Cambodian team conducted one focus group in Cambodia in the local Khmer language. The recorded session was then translated into English and back translated into Khmer so that a final English version could be analyzed across teams.

#### Ethical considerations

Protocols for consent to participate were extensively detailed for the approving Institutional Review Board at the University of Missouri-St. Louis. Oral consent for participation in the focus groups was obtained before any interaction took place and Cambodian participants also obtained consent from persons they interviewed after demonstrations by U.S. researchers. An apprenticeship model including 2 U.S. researchers supported the Cambodian researcher team (2–4 individuals) to plan the focus groups in Cambodia, and complete 1–2 sessions of 1 hour. The teams analyzed the data together, working in an integrated fashion using deidentified audio-recorded transcripts via online communication. After the first sessions in Cambodia, the local Cambodian team conducted a focus group session, which was deidentified, transcribed, translated and back-translated by the Cambodian team, then analyzed.

## Results of preparation and process

The focus groups conducted in each country comprised data that were analyzed and compared across groups and reported in a separate article, forthcoming. It should be noted that the content and interpretations provided many common features across countries, both in procedure and in substantive results. Cultural distinctions were present in findings with respect to practitioners’ assessments of mental health problems and needs based on the case studies, but the thrust of practitioners’ concerns about providing holistic, contextual care was very similar. This is important because it underscores the common human experiences that are present in the mental needs and challenges of people in widely differing cultural settings. This also illuminates the specific cultural nuances that must be recognized as part of every health care experience.

The Cambodian team demonstrated an excellent focus group procedure and ability to prepare a focus group session with all needed documentation. The session conducted entirely in Cambodia by Cambodian researchers produced a descriptive transcript and data that could be analyzed across focus group transcripts. It was clear the Cambodian team was adept in the skills of conducting the type of qualitative research we presented. This result was a collective achievement of mutual goals and expected aspect of the work of the team in the initial phases of the study.

The dissolution of the Cambodian team, due to the providers’ respective career shifts, before we could complete the analysis of their focus group with the entire team, was a disappointment. We were able to do an analysis with one U.S. researcher and the Cambodian-based co-investigator (results of interview analysis reported separately in a manuscript in preparation), but our intentions of critiquing and fostering additional interviews and analyses across the teams were not possible due to divergent career commitments of various team members. This was an unanticipated challenge, but we recognize that workforce cadences over time will inevitably include changing players. This understanding may be one of the poignant lessons learned in our work on this part of the project. The project continues with different Cambodian providers who are working on a randomized trial; the skills mutually acquired will benefit future projects for all involved.

## Discussion

The experience of working across countries illuminated some of the challenges of engaging in long-term sustainable international projects, but it also showed the value of planning and clarifying roles within and across research teams. Further, it underscored the importance of planning for quality *qualitative research* early in the project. The plan to start with qualitative assessment and engage a participatory qualitative research project allowed the team to settle into a reciprocal and collegial relationship that grounded the partnership, established ethical operative processes, and established a comfortable U. S. presence. We believe it was important to establish a genuine working relationship that would sustain the current and potential future projects, whereby each team was a giver and receiver, trust was fostered and partners on both sides would welcome ongoing research activity, together or with others as their careers progressed. That is, what we learned together would be sustainable, memorable, and applicable to future partnerships. Thus “leveraging qualitative approaches for sustainable partnerships” is a model for engaging in international research partnerships.

The changing roles and personnel of the original Cambodia research team was unanticipated and reduced some of the analytical time we had originally planned. This is an example of the differences in time and resources between the partnering groups. The cadence, in some ways set by grantor limits in terms of time and dollars, affected by personal and professional dynamics of team members, prevented consistency in personnel. However, the partnership itself is long standing, having started years ago in planning, grant-writing, and consistent engagement and it continues in an experimental study currently underway. The sustainability envisioned is really a progressive relationship between international partners that will allow multiple research and health care actions over time as long as there are key stakeholders committed to an extended relationship.

The incorporation of qualitative methodologies, especially as the partnering teams began in-person contact, provided an anchoring experience for the teams to learn together. Even as granting mechanisms allow for and restrict activities, early conversations, both casual and formal, could flourish using the methodologies initially implemented. Our methods were an entrée to the experimental study underway and can be repeated at various stages of the long-term partnership. Those engaged in the qualitative projects carry the knowledge and experience mutually gained and so important in mental health initiatives.

The practical ability on the part of the Cambodian team to plan, conduct and evaluate a focus group session using interpretive approaches was evident. This was likely a result of the careful planning and preparation of the U.S. team and the interest and focus of the Cambodian team, who prepared and conducted an excellent focus group session. The initial project was incomplete as a full teaching exercise without the analytical component, but didactic content and data collection techniques were strongly delivered and both teams were enriched with new knowledge on content and method. The analytical work can be extended in ongoing sessions with members of the current partnership.

The longevity of the project is an advantage and a limitation with respect to evaluating the initial circumscribed qualitative project. The qualitative arm of the project was conducted first and took many months. There were many exchanges with Cambodian government officials and the ancillary responsibilities of both Cambodian and U.S. teams required long-term interaction with various unexpected interruptions. There was attrition of original group members and changing environmental dynamics that impeded a planned execution of the initial qualitative teachings that are the focus of this article. The longer-term effects of this part of the project are speculated to be positive. This description of a slice of a sustained international partnership renders only a portion of methodological description. It is a representation of the ways in which teams come together for mutual benefit and scientific advancement.

The Khmer genocide is referenced yet today when Cambodian health professionals are asked about population health. The severity of the historical political trauma raises the stakes for health professionals and citizens in Cambodia and prioritizes mental health needs. The research team was acutely aware of this legacy as it is a backdrop, sometimes articulated and sometimes inferred, to understanding mental health in Cambodia. That is why a thoughtful qualitative approach was helpful in understanding the perspectives of the Cambodian people. Thus, the lessons learned from this partnership may be particularly informative for partnerships between countries with different political and socioeconomic backgrounds, highlighting the extremities of potential challenges and illuminating intersections that might otherwise be overlooked.

## Conclusion

International partnerships are challenged by influences that may be unexpected, funds that require annual renewal, policies that inhibit assimilation of interventions, and personnel who change. Addressing mental health needs anywhere is complex. The goal of reciprocity and mutual engagement is a primary responsibility for co-investigators and stakeholders, even if practical challenges surface. The use of qualitative methodologies, early and repeatedly, can facilitate relational understandings throughout the partnership. Thorough mutual planning and efficient delivery is important to mitigate the inevitable impediments that come, but a mutual commitment to the collaboration will yield long term benefits for all involved.
